# Awareness, current use of electronic cigarettes and associated smoking factors in Zhejiang Chinese adolescents

**DOI:** 10.1371/journal.pone.0224033

**Published:** 2019-10-21

**Authors:** Meng Wang, Ru-Ying Hu, Jin Pan, Hao Wang, Min Yu, Kai-Xu Xie, Wei-Wei Gong

**Affiliations:** 1 Department of NCDs Control and Prevention, Zhejiang Provincial Center for Disease Control and Prevention, Hangzhou, China; 2 Tongxiang Center for Disease Control and Prevention, Tongxiang, China; University College London, UNITED KINGDOM

## Abstract

**Objectives:**

The present study aims at examining the prevalence of awareness and current use of electronic cigarettes (e-cigarettes) among middle and high school students from Zhejiang, China. Smoking-related factors associated with e-cigarettes use will also be explored.

**Methods:**

This cross-sectional study was based on 2017 Zhejiang Youth Risk Behavior Survey. A total of 24,157 adolescents were recruited and relevant data of e-cigarettes and smoking-related factors were collected via a self-reported questionnaire. Logistic regression models were used to examine the association between e-cigarettes current use and the smoking-related factors. Odds ratios (ORs) and their 95% confidence intervals (CIs) were reported.

**Results:**

Overall, 70.61% of middle and high school students reported hearing of e-cigarettes, while only 2.15% reported using e-cigarettes in the past month. Among smoking-related factors, cigarette smoking (ever and current), use of other tobacco products, second hand smoke exposure and previous attempts to quit smoking were significantly associated with higher current e-cigarettes use in adolescents.

**Conclusions:**

These results presented high awareness of e-cigarettes while relatively low use in Chinese adolescents. Smoking-related factors were significantly associated with increased e-cigarettes use.

## Introduction

Electronic cigarettes (e-cigarettes) are handheld electronic devices simulating the experience of smoking a cigarette via heating a liquid which generates an aerosol, or vapor, that is inhaled by the user. Since being developed in China in 2003 and shortly afterwards introduced into market worldwide, awareness, ever and current use of e-cigarettes in adults have increased sharply [1]. In recent years, like for adults, rapid increase in awareness and use have been continued to be reported among adolescents, especially in Poland, Korea and the United States [2–3]. Although e-cigarettes have gained widespread popularity in general population, there are considerable concerns over the safety, efficacy for harmful reduction and smoking cessation with scientific evidence [4–5]. Given that youth population are probably the most vulnerable group to e-cigarettes [6–7] and the industry marketing is targeting them [8], public health concerns have been raised on the consequences of their high use. Studies have suggested that the inhaled nicotine solution, heavy metals, as well as glass fiber and flavoring chemicals from e-cigarettes may have potential adverse respiratory effects, contributing to the pathogenesis of respiratory symptoms and asthma in adolescents [9–11]. Recent literature also declares that e-cigarettes use by adolescents may increase the nicotine concentrations and dependence [12], which could confer risk for subsequent cigarette smoking [13–16]. Considering the popularity and potential adverse impacts on health, an understanding of the associated factors of e-cigarettes use is warranted to further prevent adolescents from initiating e-cigarettes use. In nature, e-cigarettes are novel tobacco and nicotine products, and research has indicated that factors associated with e-cigarettes use were similar to those related to cigarette smoking [17]. From this perspective, one way to prevent e-cigarettes use in adolescents is to avoid their exposure to relevant smoking factors. Among these smoking-related factors, early evidence have suggested that a history of smoking, family members and friends smoking, exposure to second hand smoke, and use of other tobacco products are significantly associated with the e-cigarettes use in adolescents [18–21]. However, to date, the effects of these smoking factors on e-cigarettes use in adolescents have not been explored in mainland China. Therefore, the primary objective of this study is to estimate the awareness and use of e-cigarettes in Chinese adolescents, and particularly to explore the associations between smoking-related factors and current e-cigarettes use.

## Materials and methods

### Study sample and data collection

The data were obtained from 2017 Zhejiang Youth Risk Behavior Survey (YRBS), which is a school-based survey assessing the risk behaviors in adolescents. The sampling procedures and participants characteristics are described in details elsewhere [22] and are thus only briefly recounted here. Totally, 24,157 middle and high school students were invited to participate, with 23,554 students participating in the survey, yielding a response rate of 97.50%. After excluding the ineligible questionnaires and subjects with missing key information of age and sex, 22,878 participants were recruited in the present study. In the process of drawing samples, a multistage, stratified cluster sampling technique was used. In the first stage, 30 counties were sampled from all 90 counties of Zhejiang Province on the basis of socioeconomic status. Then, schools in selected counties were stratified according to their levels (middle school, academic and vocational high school) and geographical positions (from west to east, from north to south). Finally, based on the number of students in each level of school, samples of classes were selected and students were invited to complete a self-administered questionnaire. The questionnaire used in the present study derived from U.S. 1991–2015 Youth Risk Behavior Surveillance System (YRBSS) and Global School-based Student Health Survey (GSHS). Without teachers present, students completed the anonymous questionnaire in the classroom independently. After finished, questionnaires were collected by the researchers. To make all the participants voluntary, parents / guardians of the selected students and the school officials were sent a written letter to inform them that a study was to be conducted to examine issues relevant to adolescent health, and given the option to refuse the students’ participation in the study. Consent was obtained from parents / guardians of the selected students to publish the collected data. Besides, all the researchers were strictly trained to protect the students’ privacy and ensure the confidentiality of the personal data. In particular, our study abided by the “Declaration of Helsinki” and was approved by the ethics committee of Zhejiang Provincial Center for Disease Control and Prevention.

### Definition of outcome variables

As the main interest, e-cigarettes awareness of participants was accessed using dichotomous (Yes or No) response to “Have you ever heard of e-cigarettes?” Participants were told that “e-cigarettes are battery-powered electronic devices simulating the experience of smoking a cigarette, which are shaped like cigarettes and usually contain nicotine. E-cigarettes current use of participants was accessed through the question: “During the past 30 days, on how many days did you use e-cigarettes? (0 day, 1–2 days, 3–5 days, 6–9 days, 10–19 days, 20–29 days, 30 days)”. Participants were considered as current users if they answered had used e-cigarettes at least 1 day during the past 30 days.

### Definition of exposure variables

The definitions of smoking-related factors were described in details in Table 1.

**Table 1:** The definition of smoking-related factors in the current study.

Smoking-related factors	Definition
Ever smoking (Yes, No)	“Have you ever tried cigarette smoking, even one or two puffs? (Yes or No).” Ever smoking was identified if they answered had tried cigarette smoking, even one or two puffs.
Current smoking (Yes, No)	“During the past 30 days, on how many days did you smoke cigarettes? (0 day, 1–2 days, 3–5 days, 6–9 days, 10–19 days, 20–29 days, 30 days)”. Participants were considered as current smoking if they answered had smoked at least 1 day during the past 30 days.
Number of cigarettes smoked (≤1, 2–10, ≥10)	“How many cigarettes did you smoke every day during the past 30 smoking days? (0, <1, 1, 2–5, 6–10, 11–20, >20)”.
Attempts to quit smoking (Yes, No)	“During the past 12 months, did you attempt to quit smoking (Yes or No)”
Exposure to second hand smoke (Yes, No)	“How many days are you exposed to the tobacco smoking exhaled by smokers over 15 minutes in a week? (Every day, >3 days, 1–3 days, <1day, 0 day)”. Passive smoking was identified if participants were exposed tobacco smoking at least 1day in a week.
Ever use of other tobacco products (Yes, No)	“Have you ever tried any other tobacco products, such as hookah, cigar, and pipe? (Yes or No).”
Living with smokers (Yes, No)	“Did you live with smokers? (Yes, No)”.
Perceived smoking as harmful (Yes, No)	“Do you think smoking is harmful to health? (Yes, No)”.

### Other covariates

Some covariates including age range (≤13, 14, 15, ≥16 years), sex (girls, boys), location of school (urban, rural), and school level (middle school, academic high school, vocational high school) were also taken into consideration in this study.

### Statistical analysis

Descriptive statistics were used to estimate the prevalence of e-cigarettes awareness and current use among middle and high school students and the prevalence in adolescents with different characteristics were compared using chi-square test and linear-by-linear association chi-square test. a P value of <0.05 was considered to be statistically significant. To explore the associated smoking factors of current use of e-cigarettes among adolescents, logistic regression was performed in three models. Note that this study used school level data and generally, the multi-level model should be adopted. However, the results from the model testing indicated that the data were not suitable for the multi-level analyses and thus, multivariable logistic regression models were conducted to adjust for the covariates in this study. Model 1 showed the results of univariate logistic regression with no potential confounders were adjusted for. Model 2 adjusted for the social-demographic characteristics including age, sex, location and level of school. Finally, Model 3 adjusted for social-demographic characteristics (age, sex, location and level of school) and smoking-related factors described in Table 1. The possible effects of smoking-related factors on current use of e-cigarettes in adolescents were showed with odds ratios (ORs) and their 95% confidence intervals (CIs). All analyses were performed using SAS statistical package (version 9.2, SAS Institute, Inc., Cary, NC, USA).

## Results

Out of 22,878 students included in the study, 1,214 (5.31%, 95% CI: 5.02%-5.60%) were current smokers. Besides, 16,149 (70.61%, 95% CI: 70.02%-71.20%) have heard of e-cigarettes and 492 (2.15%, 95% CI: 1.97%-2.35%) were current e-cigarettes users. The overall prevalence of current e-cigarettes use was relatively low, but increased significantly with smoking status and number of cigarettes smoked every day: never smokers 1.02% (95% CI: 0.88%-1.18%), ever smokers 6.67% (95% CI: 5.96%-7.42%), current smokers 17.97% (95% CI: 15.85%-20.25%); less than 1 cigarette per day 1.08% (95% CI: 0.95%-1.23%), 2–10 cigarettes per day 34.56% (95% CI: 30.65%-38.63%), more than 10 cigarettes per day 68.83% (95% CI: 57.26%-78.91%). Besides, the prevalence of current e-cigarettes use was higher in adolescents who attempted to quit smoking (17.54% vs. 8.13%), were exposed to second hand smoke (5.04% vs. 1.41%), ever used other tobacco products (47.98% vs. 1.79%), lived with smokers (2.52% vs. 0.96%), did not perceive smoking as harmful (8.50% vs. 1.98%) (all P <0.001) (Table 2).

**Table 2:** Prevalence of awareness and current use of electronic cigarettes in adolescents in Zhejiang Province, China.

Characteristics	Awareness		P	Current use		P
	Number (%)	95% CI		Number (%)	95% CI	
Total	16149/22870 (70.61)	70.02–71.20		492/22870 (2.15)	1.97–2.35	
Age groups (years)†			<0.001			0.605
≤13	1527/2729 (55.95)	54.07–57.83		60/2729 (2.20)	1.68–2.82	
14-	2170/3726 (58.24)	56.64–59.83		83/3727 (2.23)	1.78–2.75	
15-	2662/4011 (66.37)	64.88–67.83		89/4011 (2.22)	1.79–2.72	
≥16	9790/12404 (78.93)	78.20–79.64		260/12403 (2.10)	1.85–2.36	
Sex			<0.001			<0.001
Boys	8999/11655 (77.21)	76.44–77.97		392/11655 (3.36)	3.04–3.71	
Girls	7150/11215 (63.75)	62.86–64.64		100/11215 (0.89)	0.73–1.08	
Location of school			<0.001			0.001
Urban	6400/8754 (73.11)	72.17–74.04		153/8754 (1.75)	1.48–2.04	
Rural	9749/14116 (69.06)	68.29–69.83		339/14116 (2.40)	2.16–2.67	
School level†			<0.001			0.001
Middle school	7382/11887 (62.10)	61.22–62.97		265/11889 (2.23)	1.97–2.51	
Academic high school	4917/6343 (77.52)	76.47–78.54		58/6342 (0.91)	0.70–1.18	
Vocational high school	3850/4640 (82.97)	81.86–84.05		169/4639 (3.64)	3.12–4.22	
Ever smoking			<0.001			<0.001
Yes	3810/4560 (83.55)	82.44–84.62		304/4561 (6.67)	5.96–7.42	
No	12329/18297 (67.38)	66.70–68.06		187/18296 (1.02)	0.88–1.18	
Current smoking			<0.001			<0.001
Yes	1121/1213 (92.42)	90.80–93.84		218/1213 (17.97)	15.85–20.25	
No	15026/21655 (69.39)	68.77–70.01		274/21655 (1.27)	1.12–1.42	
Number of cigarettes smoked†			<0.001			<0.001
≤1 per day	15514/22221 (69.82)	69.21–70.42		241/22221 (1.08)	0.95–1.23	
2–10 per day	561/570 (98.42)	91.22–95.34		197/570 (34.56)	30.65–38.63	
≥10 per day	72/77 (93.51)	85.49–97.86		53/77 (68.83)	57.26–78.91	
Attempts to quit smoking			<0.001			<0.001
Yes	786/855 (91.93)	89.90–93.67		150/855 (17.54)	15.05–20.26	
No	773/1009 (76.61)	73.87–79.19		82/1009 (8.13)	6.51–9.99	
Exposure to second hand smoke			<0.001			<0.001
Yes	3540/4690 (75.48)	74.22–76.71		235/4690 (5.01)	4.40–5.67	
No	12601/18171 (69.35)	68.67–70.02		257/18171 (1.41)	1.25–1.60	
Ever use of other tobacco products			<0.001			<0.001
Yes	239/267 (89.51)	85.20–92.92		83/173 (47.98)	40.34–55.69	
No	15900/22583 (70.41)	69.81–71.00		407/22691(1.79)	1.62–1.97	
Living with smokers			<0.001			<0.001
Yes	12729/17531 (72.61)	71.94–73.27		441/17530 (2.52)	2.29–2.76	
No	3415/5332 (64.05)	62.74–65.34		51/5333 (0.96)	0.71–1.26	
Perceived smoking as harmful			0.547			<0.001
Yes	15727/22265 (70.64)	70.03–71.23		441/22265 (1.98)	1.80–2.17	
No	417/600 (69.50)	65.64–73.16		51/600 (8.50)	6.39–11.02	

CI: confidence interval.

^†^ Prevalence between these groups was compared using linear-by-linear association chi-square test.

Table 3 showed the specific results of univariate and multivariable logistic regression analyses in different models. Without adjusting for any covariates, model 1showed that e-cigarettes use was significantly associated with all the smoking-related factors. Ever smoking (OR: 6.29, 95% CI: 5.75–8.32), current smoking (OR: 17.10, 95% CI: 14.15–20.65), number of cigarettes smoked (OR: 0.06, 95% CI: 0.05–0.08 for 2–10 per day and OR: 0.03, 95% CI: 0.02–0.05 for ≥10 per day), attempts to quit smoking (OR: 2.41, 95% CI: 1.81–3.20), exposure to second hand smoke (OR: 3.68, 95% CI: 3.07–4.40), ever use of other tobacco products (OR: 50.49, 95% CI: 36.89–69.12), living with smokers (OR: 2.67, 95% CI: 2.00–3.58), perceived smoking as harmful (OR: 0.22, 95% CI: 0.16–0.29). Model 2 showed the results after adjusting for the social-demographic characteristics. Ever smoking (OR: 6.00, 95% CI: 4.94–7.28), current smoking (OR: 14.06, 95% CI: 11.40–17.33), number of cigarettes smoked (OR: 0.09, 95% CI: 0.07–0.12 for 2–10 per day and OR: 0.04, 95% CI: 0.03–0.07 for ≥10 per day), attempts to quit smoking (OR: 2.06, 95% CI: 1.53–2.78), exposure to second hand smoke (OR: 3.16, 95% CI: 2.63–3.80), ever use of other tobacco products (OR: 33.48, 95% CI: 24.06–46.59), living with smokers (OR: 2.36, 95% CI: 1.76–3.17), perceived smoking as harmful (OR: 0.28, 95% CI: 0.21–0.39). After adjusting for all the potential confounders, model 3 showed that e-cigarettes use in adolescents were significantly associated with ever smoking (OR: 2.12, 95% CI: 1.31–3.42), current smoking (OR: 1.88, 95% CI: 1.19–2.95), attempting to quit smoking (OR: 1.44, 95% CI: 1.02–2.04), exposure to second hand smoke (OR: 1.81, 95% CI: 1.29–2.53), ever use of other tobacco products (OR: 4.93, 95% CI: 3.24–7.50).

**Table 3:** Associations between smoking-related factors and current use of electronic cigarettes in different models.

Smoking-related factors	Number (%)	Model 1 (Unadjusted)		Model 2 (Only adjusted for social-demographic factors)		Model 3 (Adjusted for social-demographic and smoking-related factors)	
		OR	95% CI	OR	95% CI	OR	95% CI
Ever smoking							
No	187/18296 (1.02)	Ref.		Ref.		Ref.	
Yes	304/4561 (6.67)	6.92	5.75–8.32	6.00	4.94–7.28	2.12	1.31–3.42
Current smoking							
No	274/21655 (1.27)	Ref.		Ref.		Ref.	
Yes	218/1213 (17.97)	17.10	14.15–20.65	14.06	11.40–17.33	1.88	1.19–2.95
Number of cigarettes smoked							
≤1 per day	241/22221 (1.08)	Ref.		Ref.		Ref.	
2–10 per day	197/570 (34.56)	0.06	0.05–0.08	0.09	0.07–0.12	0.94	0.63–1.38
≥10 per day	53/77 (68.83)	0.03	0.02–0.05	0.04	0.03–0.07	0.89	0.46–1.73
Attempts to quit smoking							
No	82/1009 (8.13)	Ref.		Ref.		Ref.	
Yes	150/855 (17.54)	2.41	1.81–3.20	2.06	1.53–2.78	1.44	1.02–2.04
Exposure to second hand smoke							
No	257/18171 (1.41)	Ref.		Ref.		Ref.	
Yes	235/4690 (5.01)	3.68	3.07–4.40	3.16	2.63–3.80	1.81	1.29–2.53
Ever use of other tobacco products							
No	407/22691 (1.79)	Ref.		Ref.		Ref.	
Yes	83/173 (47.98)	50.49	36.89–69.12	33.48	24.06–46.59	4.93	3.24–7.50
Living with smokers							
No	51/5333 (0.96)	Ref.		Ref.		Ref.	
Yes	441/17530 (2.52)	2.67	2.00–3.58	2.36	1.76–3.17	0.78	0.42–1.42
Perceived smoking as harmful							
No	51/600 (8.50)	Ref.		Ref.		Ref.	
Yes	441/22265 (1.98)	0.22	0.16–0.29	0.28	0.21–0.39	0.64	0.38–1.08

OR: Odds ratio, CI: confidence interval

Model 1 unadjusted for any covariates; Model 2 adjusted for social-demographic characteristics including age, sex, location and level of school; Model 3 adjusted for model 2 plus the smoking-related factors in Table 1.

## Discussion

Available research on e-cigarettes in youths is relatively limited in mainland China. Our study revealed high awareness and relatively low use of e-cigarettes in Chinese adolescents. Specifically, 70.61% of middle and high school students reported hearing of e-cigarettes, which is comparable to findings from other studies. In 2015, a mobile app-based survey of 2,042 Chinese adolescents aged 12–18 years showed that 89.52% of the participants were aware of e-cigarettes, although limited by a few drawbacks in the variable measurements and data collections [23]. According to the data released by the 2014 Global Youth Tobacco Survey (GYTS) China Project, about 45% of middle school students have heard of e-cigarettes [24]. Worldwide, an updated review study after 2014 suggested that adolescents were nearing complete awareness of e-cigarettes among current non-smokers [25]. Besides, in the United States, the adolescent awareness of e-cigarettes was reported at 50.3% in a national sample [26] and 77.3%-92.0% in state samples [27–28]. Regarding the prevalence of e-cigarettes use, our results showed that only 2.15% reported using them in the past month. Consistently, the GYTS China Project also indicated that 1.2% of middle school students reported using e-cigarettes in the last 30 days [24]. Based on Youth Smoking Survey (2012–2013) with a representative sample of 45,128 secondary students, Jiang et al. reported that the prevalence of current e-cigarettes use was 1.1% in Hong Kong Chinese adolescents. However, in the study, it is noteworthy that the low response rate at school level (19%) and self-reported data may lead to the underestimation of current e-cigarettes use [29]. Worldwide, numerous studies have suggested the past 30-day use in youth continued to be high across time [26]. In the United States, relevant surveillance data showed that the prevalence of current e-cigarettes use increased from 1.5% to 20.8% in high school students, while from 0.6% to 4.9% in middle school students during 2011–2018 [30]. In Korea, e-cigarettes use among adolescents was reported to be 9.4% in 2011, with 4.7% being current users [31], which increased from 0.5% (current and past users) reported among adolescents in 2008 [32]. Generally, the awareness and use of e-cigarettes in adolescents varied across countries and regions, which may be due to different study samples and periods, as well as local e-cigarettes or tobacco regulation policy, etc. For example, up to date, the Chinese central government has yet to make a decision on how to regulate e-cigarettes [33] while e-cigarettes have been prohibited in Hong Kong [34] and classified as tobacco products in the United States [35].

In this study, we also observed higher prevalence e-cigarettes use in current smokers (17.97%) than in ever smokers (6.67%) and non-smokers (1.02%). Using data obtained from Taiwan GYTS (2014–2016), researchers found that the current e-cigarettes use increased from 9.82% to 28.68% in adolescent smokers aged 12–18 years [36], which was higher than the e-cigarettes use rate averaged over the three years [37]. Similarly, with combining survey data separately in 13 countries, a recent systematic review presented consistent higher e-cigarettes use in current smoking youth (29.9%-71.9%) than in all youth (5.9%-62.1%) and non-smoking youth (4.2%-14.0%) [2]. Besides, our findings showed that more than a third (187/492) of e-cigarettes current users was never smoking adolescents, confirming the growing concern about the high use of e-cigarettes in adolescents who never used tobacco. Recently, a UK-based survey of 16,193 school students aged 14–17 years reported that 15.8% of e-cigarettes users had never smoked conventional cigarettes [38]. More recently, another UK study conducted among 499 school pupils aged 11–16 years indicated that 52.6% of e-cigarettes users had never used tobacco [39]. Meanwhile, among adolescents in the United States, past month e-cigarettes use in both sexes increased significantly from 2011–2015 in any other tobacco non-users [40]. Given the possible “gateway effect” of e-cigarettes on tobacco use, particular surveillance of e-cigarettes use in never-smoking adolescents is warranted.

Furthermore, this study focused on identifying smoking-related factors associated with current e-cigarettes use. Consistent with previous studies [21, 41], we found that use of cigarettes (ever and current) and other tobacco products were positively associated with e-cigarettes use in adolescents. Although the mechanisms underlying the higher possibility of e-cigarettes use in tobacco products users are complicated, according to a prior review study [18], smokers generally perceived e-cigarettes as less harmful and were more willing to try e-cigarettes, especially when exposed to the e-cigarettes ads. The other tobacco products, such as waterpipe and snus, as reported previously, shared the common appeal to adolescents seeking novelty or sensation with e-cigarettes [21]. Besides, due to the susceptibility to nicotine, adolescent tobacco products users may easily produce nicotine dependence and addiction. From this view, e-cigarettes, as a good source of nicotine, may be more likely to be used by tobacco products users. Existing literature has demonstrated that second hand smoke inside or outside home was associated with increased susceptibility to any nicotine product use, including e-cigarettes [42–44]. In this study, we further reported that exposure to second hand smoke at home was significantly associated with increased e-cigarettes use in adolescents. Similarly in US adolescents, Zhang et al. also found that second hand smoke exposure at home was a significant predictor of e-cigarettes use, regardless of family smoking status [20]. However, given the evidence on the association of second hand smoke is limited and based on cross-sectional studies, more solid evidence with longitudinal design is warranted. Although promoted as a cessation product, whether e-cigarettes are actually used for smoking cessation is uncertain. In line with Korean studies [31, 45], we found that adolescents who attempted to quit smoking in the past 12 months were more likely to use e-cigarettes, indicating that e-cigarettes were being used as a cessation product. However, in another study among Chinese youth, no significant relationship was seen between having tried to stop smoking and e-cigarettes use [24]. Meanwhile, in New Zealand and the United States, previous quit attempts were also not observed to be associated with e-cigarettes use [14, 46]. To some extent, these non-significant associations coincided with the previous findings that the main reasons for trying e-cigarettes in adolescents was curiosity, independent of smoking status [46–47].

There are several limitations to this study. First, with the cross-sectional design, the causality on associations between smoking factors and e-cigarettes use could not be determined. Second, since some newer e-cigarette products not shaped like cigarettes emerged, the prevalence of e-cigarettes awareness and current use may be underestimated based on the question item used in the method. Third, as the study sample was selected at provincial level, these findings may not be generalized to all Chinese adolescents.

## Conclusions

In summary, we presented high awareness and relatively low use of e-cigarettes among middle and high school students in Zhejiang Province, China. Although the prevalence of use was relatively low, considering the large number of Chinese youth, the e-cigarettes issue would be a public health problem faced by the government. We also identified smoking-related factors that were associated with current e-cigarettes use in adolescents, including cigarette smoking (ever and current), use of other tobacco products, second hand smoke exposure and previous attempts to quit smoking. In developing health education programs and regulatory interventions, the factors aforementioned should deserve more attention.

## Supporting information

S1 File The data set used for analysis in this study. (SAV)
